# Quality of Life of Pregnant Women with Systemic Lupus Erythematosus

**DOI:** 10.1055/s-0042-1743092

**Published:** 2022-02-17

**Authors:** Larissa Rodrigues, Maria Laura Costa, Francisco Carlos Specian, Maria Margarida Fialho Sim-Sim, Fernanda Garanhani Surita

**Affiliations:** 1 Departamento de Tocoginecologia, Universidade Estadual de Campinas, Campinas, SP, Brazil; 2 Departamento de Enfermagem, Universidade de Évora, Évora, Portugal

**Keywords:** pregnancy, systemic lupus erythematosus, quality of life, gravidez, lúpus eritematoso sistêmico, qualidade de vida

## Abstract

**Objective** To assess the quality of life (QoL) of pregnant women with systemic lupus erythematosus (SLE) treated at a high-risk prenatal outpatient clinic during the third trimester of gestation.

**Methods** An observational descriptive study was performed in a high-risk prenatal outpatient clinic. Women in the third trimester of pregnancy and undergoing antenatal care between July 2017 and July 2019 answered the abbreviated World Health Organization Quality of Life (WHOQOL-BREF) questionnaire, consisting of 26 questions divided into 4 domains (physical, psychological, social and environmental).

**Results** We interviewed 50 pregnant women with a mean gestational age of 30 weeks (standard deviation [SD]: 10 weeks) who were diagnosed with SLE. The average age of the participants was 30 years (SD: 14.85), and the average time since the diagnosis of SLE was of 9.06 years (SD: 6.8 years). Most participants had a partner, did not plan their pregnancy (76%), and did not use contraception prior to pregnancy (80%). The score of each domain ranges from 0 (the worst score) to 100 (the best score). The means ±  SDs of the scores of the participants on each domain were: physical – 52.21 ±  18.44); psychological – 64.17 ±  18.56); social – 66.33 ±  27.09); and environmental – 64.56 (18.53). The means ±  SDs of the general QoL, and health-related QoL items were of 70.50 ±  24.06 and 70.00 ±  30.72 respectively.

**Conclusion** The physical domain presented the lowest scores compared with the other three domains. Pregnant women with SLE had high overall QoL scores, and their health-related QoL scores were also relatively high.

## Resumo

**Objetivo** Investigar a qualidade de vida (QV) de gestantes com lúpus eritematoso sistêmico (LES), em acompanhamento ambulatorial pré-natal de alto risco, durante o terceiro trimestre de gestação.

**Métodos** Foi realizado um estudo observacional descritivo em ambulatório de pré-natal de alto risco. As mulheres em acompanhamento pré-natal no terceiro trimestre de gravidez entre julho de 2017 e julho de 2019 responderam ao questionário abreviado de Qualidade de Vida da Organização Mundial de Saúde (abbreviated World Health Organization Quality of Life, WHOQOL-BREF, em inglês), composto por 26 questões divididas em 4 domínios (físico, psicológico, social e ambiental).

**Resultados** Foram entrevistadas 50 gestantes com diagnóstico de LES e média de 30 semanas de idade gestacional (desvio padrão [DP]: 10 semanas). A idade média das participantes foi de 30 anos (DP: 14,85), e o tempo médio desde o diagnóstico de lúpus foi de 9,06 anos (DP = 15,55 anos). A maioria das participantes tinha companheiro, não havia planejado a gravidez (76%), e não fazia uso de anticoncepcional antes da gravidez (80%). A pontuação em cada domínios varia de 0 (pior pontuação) a 100 (melhor pontuação). As médias ±  DPs das pontuações das participantes em cada domínios foram: físico – 52,21 ±  18,44; psicológico – 64,17 ±  18,56; social – 66,33 ±  27,09; e ambiental –64,56 ±  18,53). As médias ±  DPs dos itens relativos à QV geral e à QV relacionada à saude foram de 70,50 ±  24,06) e 70,00 ±  30,72, respectivamente.

**Conclusão** O domínio físico apresentou as menores pontuações em comparação com os outros três domínios. Mulheres grávidas com LES tiveram pontuação alta no item de QV geral, e a pontuação no item de QV relacionada à saúde também foi relativamente alta.

## Introduction

Systemic lupus erythematosus (SLE) is an autoimmune, multisystem disease that can cause damage to the skin, kidneys, heart, lungs, and other organs. It is more prevalent among women of reproductive age, and has been associated with a high risk of adverse maternal and perinatal outcomes. 1 2

The most common conditions observed during pregnancy include hypertension, nephropathy, and the presence of autoantibodies, which can affect the materno-fetal binomial. 3 4 The management of SLE during pregnancy can become challenging, due to difficulties distinguishing disease manifestations from the physiological changes associated with pregnancy, and due to the increased need for therapeutic control and surveillance. 5 Therefore, pregnancy may affect the quality of life (QoL) of women with SLE.

The World Health Organization 6 (WHO) defines QoL as how people perceive their position in life, how they view their goals, their expectations, and their concerns, and how they relate to the culture and values of the place where they live. It can be measured with validated instruments, such as the abbreviated World Health Organization Quality of Life (WHOQOL-BREF) questionnaire. 7

The WHOQOL-BREF has been used to measure general QoL, and it has been validated in Portuguese, 7 making this an appropriate tool for use in Brazil. The questionnaire is not specific to the functional aspects associated with health during pregnancy or with women with SLE. Although the WHOQOL-BREF has been used in Brazil for the assessment of other pregnant women undergoing usual-risk 8 9 10 and high-risk prenatal care, 8 11 12 the present study is the first to use this instrument in women with two health conditions: pregnancy and SLE.

The QoL of pregnant women with SLE has already been discussed, and their perceptions of pregnancy in the context of the disease appear to be ambiguous. Some reports 13 have described a sense of well-being and satisfaction accompanying pregnancy, in addition to fears and uncertainties regarding the limitations that the disease may impose on materno-fetal health. Women with SLE desire the experience of maternity; however, to fulfill this desire, they require adequate support. 13

Considering the need for good health practices during pregnancy, childbirth and the postpartum period, 14 15 as well as the recommendation that prenatal care is a good experience for women, 16 17 the present study aimed to assess the QoL of women with SLE during the third trimester of pregnancy while being treated at a specialized prenatal care unit.

## Methods

An observational descriptive study was conducted at Woman's Hospital Professor José Aristodemo Pinotti, State University of Campinas, Brazil, a reference in terms of health assistance to ∼ 100 municipalities in the region. The hospital has three prenatal outpatient clinics: High-Risk Prenatal Care (Pré-natal de Alto Risco, PNAR, in Portuguese), which provides care for pregannt woman with clinical and obstetric diseases; Adolescent Prenatal Care (Pré-natal de adolescentes, PNA, in Portuguese), which assists pregnant women up to 18 years of age; and the Specialized Prenatal Care (Pré-natal Especializado, PNE, in Portuguese), which assists women with more complex pregnancies, after the initial screening of the PNAR. Our research scenario was focused specifically on the PNE. Approximately 40 pregnant women are treated at this site on Wednesdays, including a yearly average of 25 SLE patients.

Pregnant women with a diagnosis of SLE (with any degree of severity) 18 were eligible if they were in the third trimester and undergoing prenatal care. The exclusion criteria were: illiterate patients and those without cognitive conditions to understand and talk about QoL. The sampling was intentional: all women who were treated at the aforementioned outpatient clinic between July 2017 and July 2019 were invited to participate in the study, and there were no cases of refusal.

The participants received an explanation about the topic and objectives of the study, and about the rights of the parties involved. The interviewer read and explained the Informed Consent Form to the participants, and the interviews were only conducted after the patients indicated their understanding and signed the form. Then, their sociodemographic data were collected (age, time since SLE diagnosis, parity, miscarriage or fetal death, planned pregnancy, previous contraception, level of schooling, occupational status, and marital status). The questionnaire was applied as an interview, and took an average of 15 minutes to administer. The participants were approached on the same day of their medical consultation for prenatal care. The interviewees were guaranteed confidentiality. Permission was requested to use a recording device.

The instrument used to analyze the QoL measurements was the WHOQOL-BREF, 7 a questionnaire with 26 items regarding QoL divided into 4 domains: physical, psychological, social, and environmental. These 26 items are presented in the format of a Likert scale, with scores from 1 to 5. Lower scores represent worse perceptions of QoL. For the proper interpretation of the results, questions Q3, Q4 (physical domain – sleep and rest and mobility), and Q26 (global QoL – satisfaction with health) have reversed scores, with 5 representing the worst score and 1 representing the best score. 19

The total score on the WHOQOL-BREF ranges from 0 to 100; therefore, the closer to 0, the worse the score, whereas the best scores are closer to 100. Research defining cutoff points for women and pregnant women has not yet been reported. We found only one study 20 that discussed cutoff points for the perception of QoL among the elderly population.

The sample size was calculated as 50 participants, based on previous studies 11 18 performed with similar groups and instruments. The procedure used was the calculation of a sample size 21 to estimate a mean, using the mathematical equation *n* = (Σ *σ* / *d* ) ^2^ , in which n is the estimated sample size, z is the percentile of the normal distribution for a significance level of 5% (z = 1.96), σ is the standard deviation (SD) extracted from the studies used as references, 11 18 and d is the maximum absolute error allowed = 5 and 6.

To describe the profile of the sample according to the studied variables, frequency tables were developed for the categorical variables (the four domains of the WHOQOL-BREF, as well as the items pertaining to general QoL and health-related QoL), with values expressed as absolute frequencies (n) and percentages (%), and the descriptive statistics were used to report all numerical variables (WHOQOL-BREF scores for each of the 26 questions), with the values expressed asmeans ±  SDs, minimum and maximum, medians, and quartiles. The normality of distribution was tested by histogram, normal-plot, and the Kolmogorv-Smirnov test. The Statistical Analysis System (SAS System for Windows, SAS Institute Inc., Cary, NC, United States) software, version 9.2, was used to perform the statistical analyses.

The present study was performed in accordance with Resolution no. 466 of the Brazilian National Health Council 22 on health research with human beings, and received authorization from the local Ethics Committee under the number #68143817.0.0000.5404.

## Results

A total of 50 pregnant women (gestational age: 30 ± 10 weeks) with SLE participated in the present study. They had an average age of 30 ± 14.85 years and an average time since SLE diagnosis of 9.06 ± 6.8 years. Most participants had a partner, did not plan their pregnancies, and did not use contraception. The sample had a normal distribution. The sociodemographic characteristics are show in Table 1 .

**Table 1:** Characteristics of the study sample

Participants ( *n* = 50)		n(%)
Variable	Categories	
Age (years)	≤ 30	27(54)
	> 30	23(46)
Time since the diagnosis of systemic lupus erythematosus (years)	≤ 5	23(46)
	> 5	27(54)
Parity	Primiparous	18(36)
	Multiparous	32(64)
Abortion or fetal death	Yes	11(22)
	No	39(78)
Planned pregnancy	Yes	12(24)
	No	38(76)
Previous contraception	Yes	10(20)
	No	40(80)
Level of schooling	High school	30(60)
	Elementary school	8(16)
	Higher education	8(16)
	No schooling	3(6)
	Technical education	1(2)
Employed	Yes	30(60)
	No	20(40)
With partner	Yes	35(70)
	No	15(30)

On each domain of the WHOQOL-BREF, our participants obtained the following mean ± SD) scores: physical – 52.21 ± 18.44); psychological – 64.17 ± 18.56); social – 66.33 ± 27.09); environmental –64.56 ± 18.53); general QoL – 70.50 ± 24.06); and health-related QoL – 70.00 ± 30.72. Table 2 shows the scores on each domain.

**Table 2:** Descriptive analysis according to the domains of the WHOQOL-bref ( *n* = 50)

Domains	n	Mean ± standard deviation	Minimum	Quartile 1	Median	Quartile 3	Maximum
Physical	50	52.21 ± 18.44	14.29	39.29	53.57	67.86	85.71
Psychological	50	64.17 ± 18.56	20.83	54.17	66.67	75.00	100.00
Social	50	66.33 ± 27.09	8.33	50.00	66.67	91.67	100.00
Environmental	50	64.56 ± 18.53	12.50	50.00	65.63	81.25	100.00
General QoL	50	70.50 ± 24.06	0.00	50.00	75.00	75.00	100.00
Health-related QoL	50	70.00 ± 30.72	0.00	50.00	75.00	100.00	100.00

Abbreviations: QoL, quality of life; WHOQOL-BREF, abbreviated World Health Organization Quality of Life questionnaire.

**Fig. 1:**
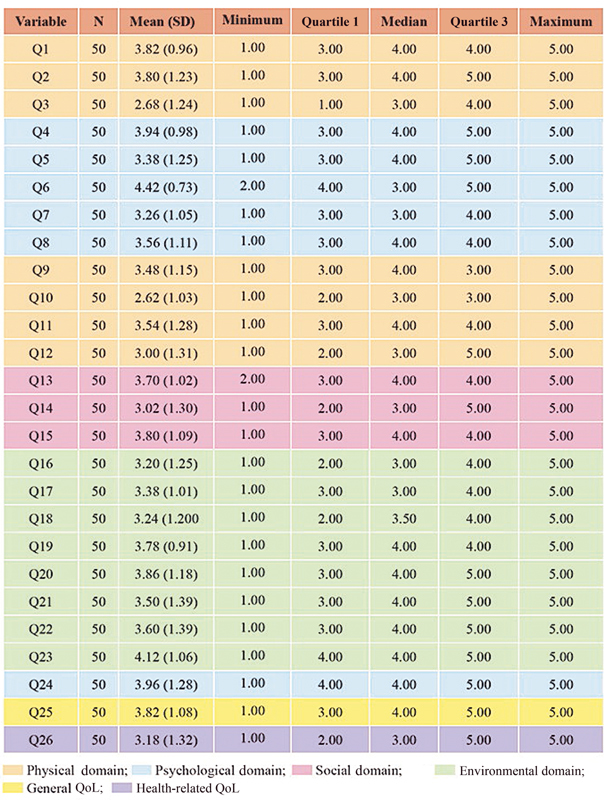
Descriptive analysis of the numerical variables ( *n* = 50).

When we examined each question of the WHOQOL-BREF, the lowest average score (2.68 ± 1.24) was found for question 3 (physical domain), which is on sleep and rest, whereas the highest average score was of 4.42 ± 0.73 for question 6 (psychological domain): “To what extent do you feel your life to be meaningful?”. Fig. 1 shows the scores on each of the 26 questions.

## Discussion

In the present study, when using the WHOQOL-BREF to asses the QoL of women with high-risk pregnancies complicated by SLE, the lowest scores were on the physical domain, with the lowest mean score reported for a question referring to activities of daily life. The scores of the participants of the present study were similar to those reported by other studies on pregnant women undergoing high-risk prenatal care. 8 23

In the present study, the physical domain of the WHOQOL-BREF was the one in which the participants scored the lowest: 52.21 ± 18.44. In another Brazilian study 8 which also evaluated pregnant women undergoing high-risk prenatal care, the physical domain was also the one with the lowest reported score (47.8 ± 15.9), and a similar finding was reported by a study conducted in Greece; 23 however, a different result was reported by a study conducted in Poland: the lowest score was on the social domain. 24

Within the physical domain, the question regarding pain and discomfort among the participants yielded an average score of 3.82, which may be associated with the control of pain issues related to SLE, either through medication or alternative therapies, which is consistent with the literature. 25 26

For the question on energy and fatigue, the participants scored an average of 3.80, which indicates that there were symptoms of fatigue that may be related to the control of the disease and its symptoms, and is similar to studies performed with non-pregnant women with SLE. 25 26

The participants scored on average 2.68 on the question regarding sleep and rest. Poor sleep quality among women with SLE, whether objective or subjective, has also been reported in a previous study. 27 Similarly, pregnant women, even without SLE, are known to have difficulties achieving good quality in terms of sleep and rest, and report symptoms of sleep disorders at significant rates. 28 Sleep disorders have also been shown to be associated with depressive symptoms. 29 30

With regard to mobility (walking, driving, climbing stairs), the participants had an average score of 3.48, which indicates a decrease in their ability to move, which may impact their autonomy, their safety, and their abilities to participate in activities, as described in a recent study 31 on the importance of mobility when caring for individuals with SLE.

Regarding the question on activities of daily living, the women had an average score of 2.62, the lowest on this domain. This suggests that women with SLE feel less capable of performing ordinary activities, which may represent the synthesis of the discomfort indicated by the other items.

With regard to dependence on medication or treatment, our subjects had an average score of 3.54, possibly due to a sense of dependence on treatments for the illness and the need to monitor the pregnancy. However, from a more subjective perspective, these women have indicated that the use of some medications makes them feel bad, and that they would prefer to focus on the pregnancy instead of the disease. 13

The analysis of the ambiguity among these reports has resulted in increasing attention being paid to the possibility that women may wish to discontinue the treatment, or are more inclined toward improved self-care while pregnant. 13

As for the question on the ability to work, the participants had an average score of 3.0, indicating that they feel less able to perform their professional activities or engage in work at home. A similar finding was reported by another study 32 regarding the difficulties encountered in relation to work after being diagnosed with SLE and the need to maintain a different routine due to the monitoring required and the limitations imposed by the disease.

The psychological score of 64.17 ± 18.56) and the environmental score of 64.56 ± 18.53 were higher than those on the other domains. In the psychological domain, a connection appears to exist between feeling healthy and being pregnant, despite the underlying presence of the disease. 13 A desire for pregnancy may also be associated with a sense of fulfillment, resulting in the sensation of psychological satisfaction.

The scores on the environmental domain are associated with the fact that the study setting coincided with the most economically- and culturally-developed region of Brazil, which is similar to the findings of other studies that have indicated that qualified insertion and good remuneration in the labor market, transportation, leisure options, and security are aspects that affect the social capital of people. 33 34

The social domain score of 66.33 ± 27.09) was the highest among the other domains in the present study; however, there are reports in the literature 32 that SLE tends to interfere with the social aspects of the affected individuals.

General QoL is assessed by a specific question, separated from the other domains, and the participants of the present study had a mean score of 70.50 ± 24.06), which is higher than the average score reported among women with SLE in other locations in Brazil, and a study 8 has reported that women with different high-risk pregnancies had an average score of 62.8 ± 13.8.

As for the assessment of health-related QoL, which is also separate from the other domains, the score of the participants of the present study was high (70 ± 30.72), but we did not find comparative values reported in other studies. This discrepancy may be associated with the access to specialized health services for the monitoring of pregnancy and childbirth among our population, as access to health services has been previously indicated and discussed as a factor that is associated with a better QoL perception. 33 34

The manifestations of SLE during pregnancy 1 3 may bother women more than usual and stand out more strongly when they refer to QoL, even when the disease is under control. Pregnancy involves major physical changes, 35 which may also be associated with the lower scores of our patients on the physical domain.

In Thessaloniki, Greece, 23 and Lublin, Poland, 24 the QoL of pregnant women diagnosed with diabetes and undergoing high-risk prenatal care has been assessed using the same instrument. The WHOQOL-BREF has also been used to assess QoL before and after the implementation of a physical exercise intervention in Brazil (in a hospital environment), and in Szczecin and Warsaw, Poland 36 (in an exercise club environment). It has also been used to assess changes in QoL among women with SLE during pregnancy and puerperium in Providence, United States. 37

In general, the QoL of pregnant women has been poorly analyzed in the literature, which has hindered the accurate assessment and establishment of adequate parameters for this population. 38

Some characteristics of our participants may be related to their perceptions of QoL. For example, 38 of the 50 women in the present study had graduated from high school or college, which means they have has more than 10 years of schooling. The literature 39 has indicated that higher levels of schooling may be associated with better perceptions of QoL. Similarly, 35 of the participants of the present study had a partner, which may be associated with the perception of social satisfaction, which is also in line with the reports in the literature. 39

Another characteristic observed among our participants is the counterpoint between the 38 women who did not plan the pregnancy and the 40 women who did not use any contraceptive method, a practically inverse relationship with the 12 who planned the pregnancy and the 10 who used a contraceptive method, which may be associated with a veiled desire for pregnancy. 13

The QoL of women diagnosed with SLE during the third trimester of pregnancy has been poorly investigated, and we have not identified any other studies on this issue. Therefore, assessing the QoL of 50 women with SLE in this scenario is unprecedented, and will contribute to future correlations regarding QoL, not only in pregnant women with lupus, but with other comorbidities during pregnancy, as many issues can be similar. The present study is limited to the assessment of QoL perceptions among women in a specific area of one country, and cannot be generalized. Future studies can contribute to the assessment of associations regarding QoL perceptions and sociodemographic characteristics of women in this area or associations with other characteristics of pregnant women with SLE in other locations. In the women herein evaluated, the main clinical complication was SLE; thus, no secondary diseases or other problems related to pregnancy were described. Another limitation is that a group of women without the disease was not recruited for comparison. This is due to the characteristics of the study site, which only assists high-risk pregnancies, and any other group would add biases to the comparison of the data.

## Conclusion

Among women with SLE treated at a specialized, high-risk prenatal care center, the physical domain of the WHOQOL-BREF had the lowest score compared with the psychological, social, and environmental domains. The pregnant women with SLE interviewed in the present study had high general QoL scores, and their health-related QoL scores were also relatively high. Certain aspects affect the QoL of these women: access to care in health services may influence the perception of health-related QoL, and the context of the region where these women live (Southeastern Brazil, the most economically-developed region of the country) may influence the social domain of the QoL.
